# Postoperative Atrial Fibrillation Impacted by Completeness of Coronary Revascularization and Antiplatelet Regimen

**DOI:** 10.1155/cdr/8857148

**Published:** 2026-02-26

**Authors:** Qin Jiang, Minghui Xie, Yalu Yu, Zhiai Tang, Jiaqi Xia, Shengshou Hu

**Affiliations:** ^1^ Department of Cardiac Surgery, Sichuan Provincial People’s Hospital, Affiliated Hospital of University of Electronic Science and Technology, Chengdu, Sichuan, China, samsph.com; ^2^ Ultrasound Medicine and Computational Cardiology Key Laboratory of Sichuan Province, Sichuan Provincial People’s Hospital, Affiliated Hospital of University of Electronic Science and Technology, Chengdu, Sichuan, China, samsph.com; ^3^ School of Medicine, University of Electronic Science and Technology, Chengdu, Sichuan, China, uestc.edu.cn; ^4^ Department of Cardiovascular Surgery, Xinqiao Hospital, The Army Medical University, Chongqing, China, xqhospital.com.cn; ^5^ Department of Cardiology, Xinjiang 474 Hospital, Urumqi, Xinjiang, China; ^6^ Department of Cardiothoracic Surgery, 363 Hospital, Chengdu, Sichuan, China; ^7^ Department of Cardiac Surgery, Fuwai Hospital, Chinese Academy of Medical Sciences and Peking Union Medical College, Beijing, China, cacms.ac.cn

**Keywords:** coronary artery bypass surgery, completeness of coronary revascularization, dual antiplatelet therapy, postoperative atrial fibrillation

## Abstract

**Objectives:**

Postoperative atrial fibrillation (POAF) is a common complication after off‐pump coronary artery bypass grafting (OPCABG) and contributes to increased morbidity and prolonged hospital stays. Both myocardial ischemia and systemic inflammation play a critical role in its pathogenesis, which is closely related to the intensity of coronary revascularization and antiplatelet treatment, respectively.

**Methods:**

This study investigated the impact of the interaction between completeness of coronary revascularization and antiplatelet regimen on POAF incidence.

**Results:**

A total of 505 eligible patients, undergoing elective first‐time OPCABG surgery from May 2017 to May 2024, were reviewed and divided into the incomplete revascularization (IR) group (*n* = 143) and complete revascularization (CR) group (*n* = 362) according to the extent of coronary revascularization. The incidence of POAF within the first week post‐OPCABG was 39.2% in the IR group versus 25.1% in the CR group (hazard ratio [HR]: 1.70, 95% confidence interval [CI]: 1.18–2.46; *p* = 0.002). AF burden (10.1% [IQR 4.8%, 16.5%] vs. 6.0% [IQR 2.3%, 9.5%], *p* = 0.003), inflammatory markers (interleukin‐6 [IL‐6] on Day 1: 104 ± 20 vs. 98 ± 16 pg/mL, *p* < 0.001), markers of prothrombotic state (D‐dimer on Day 5: 2.6 ± 0.7 mg/L FEU vs. 2.3 ± 0.7 mg/L FEU, *p* < 0.001), and postoperative hospital stay (10.7 ± 1.8 vs. 10.3 ± 1.8 days, *p* = 0.006) were significantly higher in the IR group compared to the CR group. Among patients receiving clopidogrel, POAF incidence was 42.6% (IR) vs. 25.9% (CR) (*p* = 0.001). Among patients receiving ticagrelor, POAF incidence was 25.0% (IR) vs. 22.5% (CR).

**Conclusion:**

IR was associated with a higher rate of POAF after OPCABG in patients receiving clopidogrel‐based DAPT, but not in those receiving ticagrelor‐based DAPT.

**Summary**

• **What Is Already Known on This Topic**: Postoperative atrial fibrillation (POAF) is a frequent and potentially recurrent complication occurring within the first week after off‐pump coronary artery bypass grafting (OPCABG). Myocardial ischemia and inflammation are established pathogenic drivers of POAF, which significantly increase long‐term mortality and cerebrovascular risk after isolated surgical revascularization.
• **What This Study Adds**: A total of 505 patients with three‐branch coronary stenosis undergoing elective first‐time OPCABG surgery were retrospectively reviewed and divided into incomplete revascularization (IR) group (*n* = 143), complete revascularization (CR) group (*n* = 362) according to the extent of coronary revascularization. IR was associated with significantly higher POAF incidence within 1 week after OPCABG, greater POAF burden, elevated proinflammatory cytokines, and heightened prothrombotic activity. Subgroup analysis revealed that ticagrelor‐based DAPT attenuated outcome differences between IR and CR groups—an effect absent with clopidogrel‐based DAPT.
• **How This Study Might Affect Research, Practice, or Policy**: Antiplatelet regimen selection should consider revascularization completeness to optimize POAF prevention, prioritizing ticagrelor‐based DAPT over clopidogrel in incompletely revascularized patients.

## 1. Introduction

It is well‐established that off‐pump coronary artery bypass grafting (OPCABG), compared with on‐pump CABG, is associated with favorable short‐term outcomes [1] but a higher incidence of incomplete revascularization (IR) [2]. IR has been associated with increased long‐term mortality in patients with multivessel coronary artery disease undergoing single‐artery grafting with left internal mammary artery (LIMA) [3]. Antithrombotic management is pivotal in patients with atherosclerotic cardiovascular disease. Inadequate P2Y12 inhibition and heightened adenosine diphosphate‐mediated platelet aggregation under fixed‐dose regimens are linked to increased major adverse vascular events in patients with polyvascular atherothrombotic disease [4].

POAF is a frequent and potentially recurrent complication occurring within the first week after OPCABG during intensive medical care. It disrupts normal cardiac rhythm and promotes thrombus formation and stroke risk during long‐term follow‐up [5]. Patients with POAF experiencing a high arrhythmia burden face an elevated risk of stroke and other cardiovascular events compared to those with a low burden [6]. Currently, no single modifiable risk factor reliably predicts POAF occurrence; however, an emerging AI‐based bedside tool has been developed to predict POAF with good discrimination by leveraging existing patient characteristics [7]. Nevertheless, the potential influence of specific intraoperative factors such as the completeness of revascularization combined with the postoperative antiplatelet regimen on predisposing OPCABG patients to POAF remains inadequately investigated. Therefore, we conducted this retrospective cohort study to explore how the interaction between completeness of revascularization and postoperative antiplatelet therapy influences POAF incidence and burden.

## 2. Methods

### 2.1. Ethical Approval Statement

The Medical Ethics Review Board of Sichuan Provincial People’s Hospital granted institutional approval for this study (Approval No. 2021216; March 1st, 2021) and waived the requirement for written informed consent due to the retrospective, observational design.

### 2.2. Patients

For this association analysis, we screened electronic medical records of patients undergoing elective first‐time OPCABG between May 2017 and May 2024. Inclusion criteria comprised patients who (1) underwent elective first‐time OPCABG with three‐branch stenosis and (2) received a regular single arterial graft with LIMA anastomosis to the left anterior descending (LAD) artery. Exclusion criteria included the following: preexisting atrial fibrillation/atrial flutter, uncontrolled hyperthyroidism, or multiarterial revascularization [8]. The primary outcome was the cumulative incidence of POAF within the first postoperative week. Secondary outcomes included postoperative clinical metrics during hospitalization: POAF burden, inflammatory indices, and prothrombotic markers.

### 2.3. Surgical Procedure and Definition of IR

All off‐pump coronary artery bypass anastomoses were performed via median sternotomy by an experienced surgeon under a standardized induction and anesthesia protocol. Saphenous vein grafts were harvested using conventional techniques [9] for non‐LAD targets (right coronary artery [RCA] or circumflex artery [LCx] systems). Patient‐controlled analgesia and a flexible chest tube were utilized to mitigate postoperative pain. IR was defined as the absence of a graft to any major coronary territory (RCA or LCx system) or its major branch vessels with ≥ 70% stenosis, assessed via coronary angiography review on Neusoft PACS/RIS Workstation (Version 5.5; Neusoft Corp., Shenyang, China) [10]. CR was defined as graft placement to all major coronary territories (RCA and LCx systems) with significant (≥ 70%) stenosis. If a major branch within a system (e.g., obtuse marginal branch in LCx and posterior descending artery or posterolateral branch in RCA) had significant stenosis but was not grafted, revascularization was still considered complete if another major branch within the same system received more than one graft and provided adequate flow to the ischemic territory.

### 2.4. POAF Metrics

Continuous cardiac monitoring was maintained throughout hospitalization, including ICU and intermediate care stays. Standby 12‐lead electrocardiography (ECG) was available for confirmation [11]. POAF was defined as an ECG‐documented atrial rhythm with (1) absence of distinct *P*‐waves, (2) irregular *R*–*R* intervals, and (3) ventricular rate > 100 beats/min, persisting for ≥ 30 s [12]. POAF burden was quantified per patient as follows: (1) number of distinct POAF episodes; (2) cumulative duration of all POAF episodes (hours); and (3) percentage of monitoring time in POAF [13].

### 2.5. Inflammatory Biomarkers

All patients were routinely transferred to the intensive care unit (ICU) postoperatively for monitoring and endotracheal intubation for mechanical ventilation. Antiplatelet agents were administered orally after extubation when clinically appropriate or via nasogastric tube if intubated. Beta‐blocker and statin were conventionally prescribed for all indicated patients with dosages tailored to individual conditions. Serum cytokines and simplified surrogate indicators, including interleukin‐6 (IL‐6) and neutrophil–lymphocyte ratio (NLR), were measured at three time points: preoperatively and on postoperative Days 1 and 5 [14]. Standard laboratory parameters (platelet and D‐dimer as a fibrinolytic marker) were obtained from the electronic medical record system at identical time points [15].

### 2.6. Statistical Analysis

Multivariable Cox regression analyses were performed to identify factors associated with POAF occurrence, with covariates selected based on clinical relevance and prior literature (age, sex, smoking status, obstructive sleep apnea syndrome [OSAS], and postoperative antiplatelet regimen, revascularization completeness, chronic obstructive pulmonary disease [COPD], subclinical hypothyroidism, coronary artery complexity, and anticoagulation guidance score). The cumulative incidence of POAF within the first postoperative week was analyzed using Kaplan–Meier curves with log‐rank testing. POAF burden metrics (episode count, cumulative duration, and time percentage) were analyzed using Mann–Whitney *U* tests (nonnormal distributions). Continuous variables are presented as mean ± standard deviation (SD) for normally distributed data or median with interquartile range (IQR) for nonnormally distributed data, assessed using the Kolmogorov–Smirnov test. Categorical variables are expressed as frequencies (percentage) and compared using *χ* ^2^ or Fisher’s exact tests as appropriate. All analyses were conducted using IBM SPSS Statistics (Version 25.0; Armonk, NY) and GraphPad Prism (Version 10.4.1; San Diego, CA), with statistical significance defined as two‐sided *p* < 0.05.

## 3. Results

**Figure 1:**
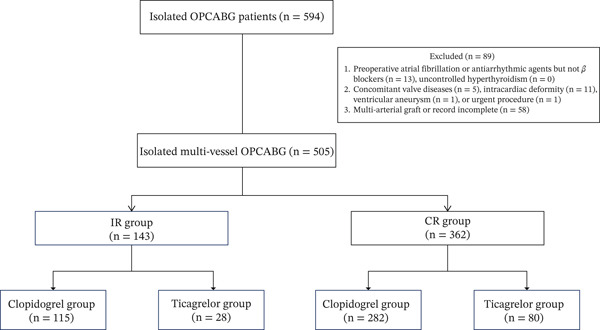
The study protocol OPCABG, off‐pump coronary artery bypass grafting; IR, incomplete revascularization; CR, complete revascularization.

After screening 594 patients against inclusion criteria, 89 were excluded. Ultimately, 505 patients were included in the final analysis and stratified into IR (*n* = 143, 28.3%) and CR (*n* = 362, 71.7%) groups (Figure 1). Baseline demographic characteristics and comorbidities were comparable between groups (Table 1). Significant intergroup differences were observed in mean number of grafts per patient (2.7 ± 0.5 vs. 3.2 ± 0.6, *p* < 0.001) and distal anastomoses per patient (2.8 ± 0.4 vs. 3.8 ± 0.6, *p* < 0.001). However, intraoperative transit‐time flow measurements demonstrated comparable graft patency between groups (Table 2).

**Table 1:** The demographic features and clinical variables.

	IR group (*n* = 143)	CR group (*n* = 362)	*p* value
Demographic characteristics			
Age (years)	56.7 ± 8.2	55.9 ± 7.8	0.343
Male, no. (%)	110 (76.9)	292 (80.6)	0.347
BMI (kg/m^2^)	23.7 ± 1.8	23.6 ± 2.0	0.767
CYP2C19 metabolizer status			0.553
Wild type	59 (41.3)	139 (38.4)	
Loss‐of‐function type	84 (58.7)	223 (61.6)	
Clinical diseases, no. (%)			
Previous MI	20 (14.0)	55 (15.2)	0.783
Previous PCI	14 (9.8)	32 (8.8)	0.733
Left main stenosis	20 (14.0)	58 (16.0)	0.682
Tobacco use, no. (%)			0.883
Never	78 (54.5)	203 (56.1)	
> 6 months ago	42(29.4)	107 (29.5)	
<6 months ago	23(16.1)	52 (14.4)	
Comorbidities profile, no. (%)			
Hypertension	82 (57.3)	203 (56.1)	0.796
Diabetes mellitus	32 (22.4)	87 (24.0)	0.693
Hypercholesterolemia	39 (27.3)	109 (30.1)	0.588
Triglyceride	28 (19.6)	68 (18.8)	0.900
OSAS	21(14.7)	42(11.6)	0.345
COPD	24(16.8)	47(13.0)	0.268
Subclinical hypothyroidism	11(7.7)	32(8.8)	0.677
Preoperative medications, no. (%)			
Aspirin	133 (93.0)	343 (94.8)	0.524
Statins	135 (94.4)	339 (93.6)	0.839
Calcium channel antagonist	45 (31.5)	125 (34.5)	0.532
Nitrate ester	88 (61.5)	211 (58.3)	0.547
Beta‐blocker	101 (70.7)	266 (73.5)	0.509
Diuretics	25 (17.5)	66 (18.2)	0.898
Cardiac function			
LAD (mm)	35.4 ± 5.3	36.9 ± 5.9	0.007
LVEDD (mm)	51.7 ± 5.7	51.4 ± 6.2	0.683
LVEF (%)	53.6 ± 10.0	54.0 ± 10.4	0.678
LVEF ≤ 35*%*, no. (%)	11 (7.7)	41 (11.3)	0.258
SYNTAX score	28.6 ± 3.2	28.4 ± 3.3	0.595
CHADS‐VASc score	2.0 ± 1.1	1.8 ± 1.1	0.085
HATCH score	1.5 ± 1.2	1.3 ± 1.2	0.187

*Note:* CHADS‐VASc score: congestive heart failure, hypertension, age ≥ 75 y (doubled), diabetes mellitus, stroke (doubled)‐vascular disease, age 65–74 and sex category (female); HATCH score, hypertension, age ≥ 75 years, transient ischemic attack/stroke, chronic obstructive pulmonary disease, and heart failure.

Abbreviations: BMI, body mass index; COPD: chronic obstructive pulmonary disease; CR, complete revascularization; CYP2C19: cytochrome P450, Family 2, subfamily C, polypeptide 19; IR, incomplete revascularization; LAD, left atrial diameter; LVEDD, left ventricular end‐diastolic dimension; LVEF, left ventricular ejection fraction; MI, myocardial infarction; OSAS, obstructive sleep apnea syndrome; PCI, percutaneous coronary intervention; SYNTAX: synergy between percutaneous coronary intervention with taxus and cardiac surgery.

**Table 2:** Intraoperative and postoperative outcomes.

	IR group (*n* = 143)	CR group (*n* = 362)	*p* value
Intraoperative conditions			
Operation time (min)	298 ± 40	330 ± 44	< 0.001
IABP assistance support, no. (%)	27 (18.9)	70 (19.3)	1.0
Graft characteristics			
Graft numbers (*n*)	2.7 ± 0.5	3.2 ± 0.6	< 0.001
Distal anastomosis number (*n*)	2.8 ± 0.4	3.8 ± 0.6	< 0.001
Transit‐time flow meter (mL/min)			
LIMA	34 ± 12	34 ± 12	0.930
D1	59 ± 22	63 ± 24	0.114
OM1/2	42 ± 12	47 ± 13	0.047
RA/PDA/PLV	36 ± 11	35 ± 10	0.384
Postoperative medications, no. (%)			0.630
Clopidogrel	115 (80.4)	282 (77.9)	
Ticagrelor	28 (19.6)	80 (22.1)	
Antiplatelet time (h)	16.7 ± 5.9	17.0 ± 5.7	0.572
ICU recovery			
Mechanical ventilation time (h)	13.6 ± 10.6	13.3 ± 8.4	0.699
Duration in ICU stay (d)	1.6 ± 0.8	1.7 ± 0.9	0.257
Re‐exploration, no. (%)	3 (2.1)	5 (1.4)	0.693
Chest drainage (mL)	774 ± 120	777 ± 120	0.829
30‐day recovery			
Death, no. (%)	1 (0.7)	2 (0.6)	1.0
Cognitive dysfunction, no. (%)	16 (11.2)	20 (5.5)	0.034
Ischemic stroke, no. (%)	6 (4.2)	3 (0.8)	0.018
Postoperative hospital stay (d)	10.7 ± 1.8	10.3 ± 1.8	0.006

Abbreviations: ACEI/ARB, angiotensin‐converting enzyme inhibitors/angiotensin receptor blockers; CCB, calcium channel blocker; CR, complete revascularization; D1, diagonal Branch 1; IABP, intra‐aortic balloon pump; IR, incomplete revascularization; LAD, left anterior descending; OM, obtuse marginal branch; PDA, posterior descending artery; PLV, posterior left ventricle.

### 3.1. POAF incidence

**Figure 2:**
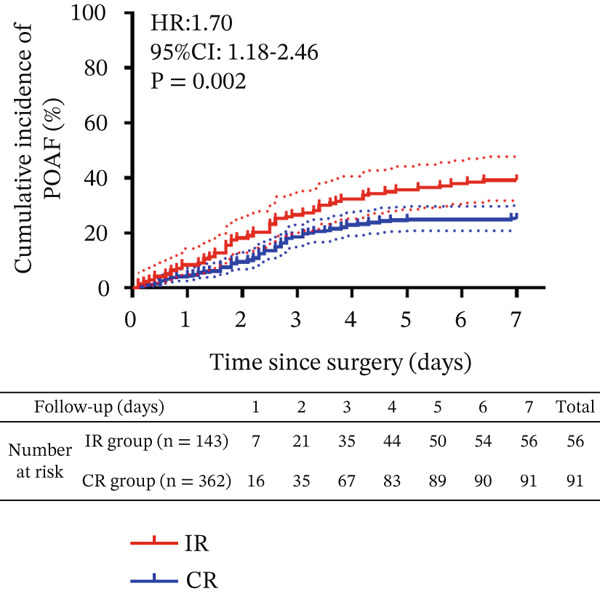
Kaplan–Meier analysis of POAF incidence by revascularization completeness. The cumulative incidence of POAF was 39.2% in the incomplete revascularization (IR) group versus 25.1% in the complete revascularization (CR) group (hazard ratio [HR] for IR vs. CR: 1.70; 95% confidence interval [CI]: 1.18–2.46; *p* = 0.002). Dashed lines represent 95% CIs. Numbers at risk are shown below the *x*‐axis. Abbreviations: POAF, postoperative atrial fibrillation; HR, hazard ratio; CI, confidence interval; IR, incomplete revascularization; CR, complete revascularization.

Multivariate Cox regression analyses identified independent prognostic factors with statistical significance only including postoperative antiplatelet regimen, revascularization completeness, and COPD. Multivariable logistic regression revealed that IR was associated with a 3.54‐fold increased risk of POAF (adjusted odds ratio [aOR] = 1.599, 95% CI: 1.141–2.241) compared to CR. Similarly, a clopidogrel‐based regimen was associated with higher POAF risk (aOR = 2.077, 95% CI: 1.309–3.293) versus ticagrelor therapy, particularly among CYP2C19 loss‐of‐function allele carriers. While, COPD imposed a modest impact on POAF (COPD: 27/71 (38.0%) vs. without COPD: 120/434 (27.6%), *p* = 0.074). Cumulative incidence analysis demonstrated significantly higher POAF occurrence in IR patients (39.2%, 56/143) versus CR patients (25.4%, 92/362), with an overall hazard ratio of 1.70 (95% CI: 1.18–2.46; *p* = 0.002; Figure 2). Time to dual antiplatelet therapy (DAPT) initiation post‐OPCABG was comparable between regimens. Stratified analysis by antiplatelet regimen: clopidogrel group: POAF incidence 42.6% (IR) vs. 25.9% (CR) (*p* = 0.001); ticagrelor group: POAF incidence 25.0% (IR) vs 22.5% (CR) (*p* > 0.05). A significant interaction between revascularization status and antiplatelet regimen was observed (*p* interaction < 0.001).

### 3.2. Inflammatory and Prothrombotic Profiles

**Figure 3:**
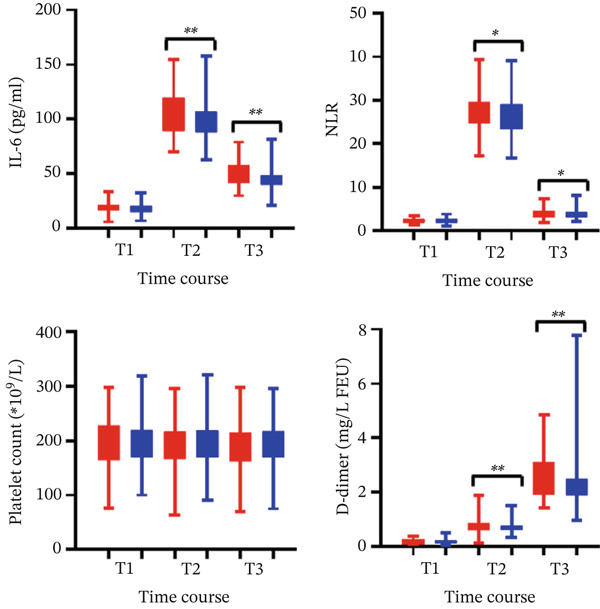
Serial measurements of inflammatory and prothrombotic markers in two groups. Levels of interleukin‐6 (IL‐6), neutrophil–lymphocyte ratio (NLR), platelet count, and D‐dimer (mg/L FEU) were measured preoperatively (T1) and on postoperative Day 1 (T2) and Day 5 (T3). Data presented as box plots (boxes: interquartile range; horizontal line: median; whiskers: range).  ^∗^ *p* < 0.05 and  ^∗∗^ *p* < 0.01 for IR vs. CR at each timepoint. *Notes:* Red bar: incomplete revascularization; blue bar: complete revascularization.

Significantly higher IL‐6 levels were observed in the IR group versus CR group at postoperative 1 day (104 ± 20 vs. 98 ± 16 pg/mL, *p* < 0.001) and 5 days (49 ± 11 vs. 45 ± 9 pg/mL, *p* < 0.001). Similarly, NLR was elevated in IR patients at postoperative 1 day (27.3 ± 3.7 vs. 26.3 ± 4.1, *p* = 0.013), and 5 days (4.0 ± 1.1 vs. 3.8 ± 1.1, *p* = 0.043). Platelet counts showed no significant intergroup differences at any measurement point. D‐dimer levels—reflecting prothrombotic activity—increased progressively in both cohorts but remained significantly higher in the IR group at postoperative 1 day (0.8 ± 0.3 mg/L FEU vs. 0.7 ± 0.2 mg/L FEU, *p* = 0.001), at postoperative 5 days (2.6 ± 0.7 mg/L FEU vs. 2.3 ± 0.7 mg/L FEU, *p* < 0.001; Figure 3).

### 3.3. Postoperative Outcomes

Length of hospital stay was significantly longer in the IR group versus CR group (10.7 ± 1.8 vs. 10.3 ± 1.8 days; *p* = 0.006; Table 2). During POAF episodes, the median ventricular rate was significantly higher in IR patients (134 [118, 145] vs. 127 [116, 133] bpm; *p* = 0.001). POAF burden metrics consistently demonstrated greater severity in the IR group: monitoring time in POAF: 10.1% [4.8%–16.5%] versus 6.0% [2.3%–9.5%] (*p* = 0.003); prolonged episodes (> 24 h): 30.4% versus 14.3% (*p* = 0.022; Table 3).

**Table 3:** POAF characteristics metrics. POAF, postoperative atrial fibrillation; IR, incomplete revascularization; CR, complete revascularization.

	IR group (*n* = 143)	CR group (*n* = 362)	*p* value
POAF incidence (*n*)	56	91	0.002
Time to onset (d)	2.5 ± 1.6	2.4 ± 1.3	0.677
Percentage of monitoring time in POAF	10.1% (4.8%, 16.5%)	6.0% (2.3%, 9.5%)	0.003
Number of distinct POAF episodes	9 (6, 14)	6 (3, 9)	< 0.001
Mean ventricular rate at POAF episodes	134 (118, 145)	127 (116, 133)	0.001
Cumulative duration of all POAF episodes (h)	17 (8, 28)	10 (4, 16)	0.003
Number of prolonged episodes			0.022
Less or equal to 24 h	39	78	
> 24 h	17	13	

## 4. Discussion

This retrospective cohort study demonstrates two principal findings: (1) IR independently increased POAF risk after OPCABG compared to CR, manifesting as higher POAF burden, amplified inflammatory response, and enhanced prothrombotic activity and (2) the POAF risk disparity between IR and CR was confined to clopidogrel‐treated patients with no significant difference observed under ticagrelor therapy, indicating a critical drug–revascularization interaction particularly pronounced in CYP2C19 loss‐of‐function allele carriers.

Personalized management by multidisciplinary critical care teams is essential given its unpredictable nature and challenges in POAF prevention [16]. Both myocardial ischemia and inflammation are established pathogenic drivers of POAF [17] with peak burden occurring on postoperative Days 2–3 [18], which significantly increases long‐term mortality and cerebrovascular risk after isolated surgical revascularization [19]. Thus, elucidating the impact of modifiable perioperative factors such as particularly revascularization completeness and antiplatelet regimen selection on POAF occurrence and severity is clinically imperative for optimizing OPCABG outcomes.

CR remains the surgical gold standard for optimizing long‐term outcomes and cardiac function in CABG [20]. In patients with multivessel disease, grafting ≥ 1 distal anastomosis per major coronary branch significantly improves survival and reduces cardiac mortality [21]. While IR of nondominant branches may not impact major adverse events, CR of all angiographically significant lesions (≥ 70% stenosis) demonstrably lowers adverse event rates [22]. However, CR achievement is frequently constrained by diffuse disease, technical limitations, and patient comorbidities [23]. Notably, contemporary data suggest IR rates are increasing over time [24]. Despite advantages in select populations like hybrid revascularization [25], OPCABG faces inherent technical challenges: hemodynamic instability during heart positioning, limited visualization of inferior/posterolateral territories, and restricted anastomotic access during beating heart surgery. Thus, mitigating the long‐term sequelae of unavoidable IR, particularly its association with heightened inflammation, POAF risk, and thrombotic burden represents a critical unmet need in surgical coronary revascularization.

IL‐6, a cytokine rapidly synthesized following coronary artery bypass grafting, orchestrates key inflammatory pathways that potentiate atherosclerotic progression. Through stimulation of endothelial activation, vascular smooth muscle proliferation, and leukocyte recruitment, IL‐6 mediates plaque destabilization and perioperative myocardial injury [26]. Simultaneously, OPCABG induces significant activation of the coagulation cascade and compensatory fibrinolysis. Notably, this prothrombotic state persists despite therapeutic heparinization in completely revascularized patients, suggesting intrinsic limitations in pharmacologic reversal of surgically triggered thrombosis [27].

Platelets are central mediators of thromboinflammation. Clinical evidence demonstrates ticagrelor’s superior net clinical benefit over other P2Y_12_ inhibitors across indications, particularly in acute coronary syndromes, high thrombotic risk profiles, and extended secondary prevention [28]. Mechanistically, ticagrelor inhibits equilibrative nucleoside transporter‐1, increasing extracellular adenosine concentrations. This potentiates adenosine’s cardioprotective effects through coronary vasodilation, anti‐inflammatory modulation, and platelet inhibition [29]. Critically, potent P2Y_12_ inhibition with ticagrelor reduces major adverse cardiovascular events in high‐risk acute myocardial infarction and cardiogenic shock patients without increasing severe bleeding risk, excluding those aged > 75 years or requiring VA‐ECMO support [30].

Patients on the clopidogrel but not ticagrelor with IR demonstrated significantly higher POAF burden and prolonged hospitalization compared to CR. This result was similar to the scenario of percutaneous coronary intervention treatment on oral clopidogrel regimen that clinical factors (such as age and acute coronary syndrome) may be more decisive than CYP2C19 genotype on the accidence rate of major adverse cardiac event [31]. Thus, these findings underscore the critical need for multidisciplinary collaboration (surgeons, intensivists, and nursing teams) to implement precision antiplatelet strategies that mitigate IR‐related risks. It was advised for IR patients to prioritize ticagrelor over clopidogrel for POAF prophylaxis in IR patients, particularly CYP2C19 LOF carriers, initiate early DAPT upon hemodynamic stabilization post‐OPCABG, and protocolize POAF screening during peak burden days. This optimized approach addresses the clinical and economic burdens of prolonged hospitalization [32] while improving outcomes for patients with residual stenosis. For unavoidable IR cases, targeted antiplatelet management represents a feasible strategy to counterbalance anatomic limitations.

## 5. Study Limitations

This analysis inherits limitations inherent to retrospective studies, including potential documentation heterogeneity in POAF detection. Despite adopting standardized criteria (ECG‐confirmed AF ≥ 30 s [33]), variations in monitoring protocols across clinicians and shifts may have resulted in under‐ascertainment of transient arrhythmias. We defined completeness of revascularization anatomically (grafting vessels with ≥ 70% stenosis) rather than functionally [34]. While anatomic assessment remains the surgical gold standard [23], this approach may overlook territories with functionally significant ischemia in moderately stenotic lesions (40%–70%), potentially attenuating observed CR benefits. Prothrombotic states were assessed via D‐dimer rather than viscoelastic testing, which could have provided mechanistic insights into platelet–fibrin interactions and hypercoagulable phenotypes not captured by conventional assays [35].

## 6. Conclusions

This real‐world analysis demonstrates that IR significantly increases POAF risk after OPCABG, particularly in clopidogrel‐treated patients where POAF incidence nearly doubled. Crucially, this risk was abrogated by ticagrelor therapy, indicating that precision antiplatelet selection mitigates IR‐related arrhythmogenicity. These findings illuminate the thromboinflammatory pathogenesis of POAF and support protocolized ticagrelor use as a preventive strategy in IR patients, especially CYP2C19 loss‐of‐function carriers.

## Author Contributions

Q.J. and Y.Y. performed the research and wrote the manuscript. Q.J. and S.H. designed the research. Q. J. and Z.T. analyzed the data. M.X. and J.X. contributed new analytical tools.

## Funding

This study was supported by the Sichuan Provincial People’s Hospital, SY2022017, and National Natural Science and Technology Foundation of China, 81800274.

## Conflicts of Interest

The authors declare no conflicts of interest.

## Data Availability

The data that support the findings of this study are available on request from the corresponding author. The data are not publicly available due to privacy or ethical restrictions.

## References

[1] Dąbrowski E. J. , Kurasz A. , Pasierski M. , Pannone L. , Kołodziejczak M. M. , Raffa G. M. , Matteucci M. , Mariani S. , de Piero M. E. , la Meir M. , Maesen B. , Meani P. , McCarthy P. , Cox J. L. , Lorusso R. , Kuźma Ł. , Rankin S. J. , Suwalski P. , Kowalewski M. , and Thoracic Research Centre , Surgical Coronary Revascularization in Patients With Underlying Atrial Fibrillation: State-of-the-Art Review, Mayo Clinic Proceedings. (2024) 99, no. 6, 955–970, 10.1016/j.mayocp.2023.12.005, 38661599. 38661599

[2] Razavi A. A. , Malas J. , Salam A. , Emerson D. A. , and Bowdish M. E. , Off-Pump Coronary Artery Bypass Grafting Is Overutilized, Seminars in Thoracic and Cardiovascular Surgery. (2025) 37, no. 1, 43–47, 10.1053/j.semtcvs.2024.12.001, 39730082. 39730082 PMC12077573

[3] Aboul-Hassan S. S. , Awad A. K. , Stankowski T. , Perek B. , Marczak J. , Rodzki M. , Jemielity M. , Moskal L. , Sá M. P. , Torregrossa G. , Gaudino M. , and Cichon R. , Impact of Incomplete Revascularization on Long-Term Survival Based on Revascularization Strategy, Annals of Thoracic Surgery. (2024) 118, no. 3, 605–614, 10.1016/j.athoracsur.2024.04.032, 38777249. 38777249

[4] Chaudhary R. and Neal M. D. , Invited Commentary: Platelet and Clot Characteristics to Guide Antithrombotic Management in Patients With Polyvascular Disease, Journal of the American College of Surgeons. (2023) 236, no. 3, 504–505, 10.1097/XCS.0000000000000493, 36729750. 36729750

[5] Benedetto U. , Gaudino M. F. , Dimagli A. , Gerry S. , Gray A. , Lees B. , Flather M. , Taggart D. P. , and Investigators A. R. T. , Postoperative Atrial Fibrillation and Long-Term Risk of Stroke After Isolated Coronary Artery Bypass Graft Surgery, Circulation. (2020) 142, no. 14, 1320–1329, 10.1161/CIRCULATIONAHA.120.046940, 33017213. 33017213 PMC7845484

[6] Linz D. , Andrade J. G. , Arbelo E. , Boriani G. , Breithardt G. , Camm A. J. , Caso V. , Nielsen J. C. , De Melis M. , De Potter T. , Dichtl W. , Diederichsen S. Z. , Dobrev D. , Doll N. , Duncker D. , Dworatzek E. , Eckardt L. , Eisert C. , Fabritz L. , Farkowski M. , Filgueiras-Rama D. , Goette A. , Guasch E. , Hack G. , Hatem S. , Haeusler K. G. , Healey J. S. , Heidbuechel H. , Hijazi Z. , Hofmeister L. H. , Hove-Madsen L. , Huebner T. , Kääb S. , Kotecha D. , Malaczynska-Rajpold K. , Merino J. L. , Metzner A. , Mont L. , Ng G. A. , Oeff M. , Parwani A. S. , Puererfellner H. , Ravens U. , Rienstra M. , Sanders P. , Scherr D. , Schnabel R. , Schotten U. , Sohns C. , Steinbeck G. , Steven D. , Toennis T. , Tzeis S. , van Gelder I. C. , van Leerdam R. H. , Vernooy K. , Wadhwa M. , Wakili R. , Willems S. , Witt H. , Zeemering S. , and Kirchhof P. , Longer and Better Lives for Patients With Atrial Fibrillation: The 9th AFNET/EHRA Consensus Conference, Europace. (2024) 26, no. 4, 10.1093/europace/euae070. PMC11003300 38591838

[7] Ma H. , Chen D. , Lv W. , Liao Q. , Li J. , Zhu Q. , Zhang Y. , Deng L. , Liu X. , Wu Q. , Liu X. , and Yang Q. , Performance of an AI Prediction Tool for New-Onset Atrial Fibrillation After Coronary Artery Bypass Grafting, EClinicalMedicine. (2025) 81, 103131, 10.1016/j.eclinm.2025.103131. 40093989 PMC11908608

[8] Jiang Q. , Huang K. , Lin S. , Wang D. , Tang Z. , and Hu S. , Impact of Multiarterial Versus Single Arterial Coronary Bypass Graft Surgery on Postoperative Atrial Fibrillation, American Journal of Cardiology. (2025) 234, 30–37, 10.1016/j.amjcard.2024.10.004, 39447720. 39447720

[9] Jiang Q. , Yang Y. , Sun H. , Tang Y. , Lv F. , and Hu S. , Stable Hemodynamics Within “No-Touch” Saphenous Vein Graft, Annals of Thoracic and Cardiovascular Surgery. (2020) 26, no. 2, 88–94, 10.5761/atcs.oa.19-00156, 31611499. 31611499 PMC7184034

[10] Jiang Q. , Du J. , Lei Y. , Gu C. , Hong L. , and Hu S. , The Relationship Between False-Lumen Area Ratio and Renal Replacement Therapy After Acute Aortic Dissection Repair on Bilateral Artery Cannulation: A Cross-Sectional Study, Quantitative Imaging in Medicine and Surgery. (2023) 13, no. 5, 3104–3114, 10.21037/qims-22-1103, 37179912. 37179912 PMC10167435

[11] Perezgrovas-Olaria R. , Alzghari T. , Rahouma M. , Dimagli A. , Harik L. , Soletti G. J. , An K. R. , Caldonazo T. , Kirov H. , Cancelli G. , Audisio K. , Yaghmour M. , Polk H. , Toor R. , Sathi S. , Demetres M. , Girardi L. N. , Biondi-Zoccai G. , and Gaudino M. , Differences in Postoperative Atrial Fibrillation Incidence and Outcomes After Cardiac Surgery According to Assessment Method and Definition: A Systematic Review and Meta-Analysis, Journal of the American Heart Association. (2023) 12, no. 19, e030907, 10.1161/JAHA.123.030907. 37776213 PMC10727249

[12] Jiang Q. , Liu S. Z. , Jiang L. , Huang K. L. , Guo J. , and Hu S. S. , Comparison of Two Radiofrequency Ablation Devices for Atrial Fibrillation Concomitant With a Rheumatic Valve Procedure, Chinese Medical Journal. (2019) 132, no. 12, 1414–1419, 10.1097/CM9.0000000000000276, 2-s2.0-85068240705, 31205098. 31205098 PMC6629330

[13] Jiang Q. , Huang K. , Yin L. , Kong H. , Yang Z. , and Hu S. , Effect of Ticagrelor Versus Clopidogrel After Off-Pump Coronary Artery Bypass Grafting on Postoperative Atrial Fibrillation: A Cohort Study, Journal of the American Heart Association. (2024) 13, no. 16, e035424, 10.1161/JAHA.124.035424. 39140333 PMC11963955

[14] Jiang Q. , Yu T. , Huang K. L. , Liu K. , Li X. , and Hu S. S. , Carotid Versus Axillary Artery Cannulation for Descending Aorta Remodeling in Type A Acute Aortic Dissection, World Journal of Cardiology. (2024) 16, no. 10, 564–573, 10.4330/wjc.v16.i10.564, 39492974. 39492974 PMC11525798

[15] Jiang Q. , Li H. , Huang X. , Yu L. , Lueck S. , and Hu S. , Postnatal Exposure to Hypobaric Hypoxia and Its Impact on Inflammation and Injury Indexes After a Cardiac Valve Procedure, Interactive Cardiovascular and Thoracic Surgery. (2020) 31, no. 6, 789–795, 10.1093/icvts/ivaa188, 33118008. 33118008

[16] Sibley S. , Bedford J. , Wetterslev M. , Johnston B. , Garside T. , Kanji S. , Whitehouse T. , Welters I. , Ostermann M. , Balik M. , Lancini D. , Dharmaraj B. , Benjamin E. J. , Walkey A. J. , and Cuthbertson B. H. , Atrial Fibrillation in Critical Illness: State of the Art, Intensive Care Medicine. (2025) 51, no. 5, 904–916, 10.1007/s00134-025-07895-0, 40323451. 40323451

[17] Gaudino M. , Di Franco A. , Rong L. Q. , Piccini J. , and Mack M. , Postoperative Atrial Fibrillation: From Mechanisms to Treatment, European Heart Journal. (2023) 44, no. 12, 1020–1039, 10.1093/eurheartj/ehad019, 36721960. 36721960 PMC10226752

[18] Perezgrovas-Olaria R. , Chadow D. , Lau C. , Rahouma M. , Soletti G. J. , Cancelli G. , Harik L. , Dimagli A. , Rong L. Q. , Gillinov M. , Ad N. , DiMaio M. , Gelijns A. C. , Sanna T. , Fremes S. , Crea F. , Girardi L. , and Gaudino M. , Characteristics of Postoperative Atrial Fibrillation and the Effect of Posterior Pericardiotomy, Annals of Thoracic Surgery. (2023) 116, no. 3, 615–622, 10.1016/j.athoracsur.2022.11.007, 36375495. 36375495 PMC10468100

[19] Oraii A. , Masoudkabir F. , Pashang M. , Jalali A. , Sadeghian S. , Mortazavi S. H. , Ghorbanpour Landy M. , Pourhosseini H. , Salarifar M. , Mansourian S. , Bagheri J. , Momtahan S. , and Karimi A. , Effect of Postoperative Atrial Fibrillation on Early and Mid-Term Outcomes of Coronary Artery Bypass Graft Surgery, European Journal of Cardio-Thoracic Surgery. (2022) 62, no. 3, 10.1093/ejcts/ezac264. 35441680

[20] Cho H. , Sohn S. H. , and Hwang H. Y. , Contemplation Behind the Definition of Complete Revascularization, Annals of Thoracic Surgery. (2025) 119, no. 4, 923–924, 10.1016/j.athoracsur.2024.11.027, 39706509. 39706509

[21] Cho H. , Kim J. S. , Kang Y. , Sohn S. H. , and Hwang H. Y. , Impact of More Than 1 Distal Anastomosis on the Same Territory in 3-Vessel Disease Patients, Annals of Thoracic Surgery. (2025) 119, no. 3, 546–554, 10.1016/j.athoracsur.2024.09.038, 39396670. 39396670

[22] Bianco V. , Kilic A. , Aranda-Michel E. , Serna-Gallegos D. , Ferdinand F. , Dunn-Lewis C. , Wang Y. , Thoma F. , Navid F. , and Sultan I. , Complete Revascularization During Coronary Artery Bypass Grafting Is Associated With Reduced Major Adverse Events, Journal of Thoracic and Cardiovascular Surgery. (2023) 166, no. 1, 104–113.e5, 10.1016/j.jtcvs.2021.05.046, 34272071. 34272071

[23] Gaba P. , Gersh B. J. , Ali Z. A. , Moses J. W. , and Stone G. W. , Complete Versus Incomplete Coronary Revascularization: Definitions, Assessment and Outcomes, Nature Reviews Cardiology. (2021) 18, no. 3, 155–168, 10.1038/s41569-020-00457-5, 33067581. 33067581

[24] Soukup C. R. , Schmidt C. W. , Chan-Tram C. , Garberich R. F. , Sun B. C. , and Traverse J. H. , Rate of Incomplete Revascularization Following Coronary Artery Bypass Grafting at a Single Institution Between 2007 and 2017, American Journal of Cardiology. (2021) 144, 33–36, 10.1016/j.amjcard.2020.12.064, 33383011. 33383011

[25] Besola L. , Colli A. , and De Caterina R. , Coronary Bypass Surgery for Multivessel Disease After Percutaneous Coronary Intervention in Acute Coronary Syndromes: Why, for Whom, How Early?, European Heart Journal. (2024) 45, no. 34, 3124–3131, 10.1093/eurheartj/ehae413, 39056269. 39056269

[26] Brull D. J. , Montgomery H. E. , Sanders J. , Dhamrait S. , Luong L. , Rumley A. , Lowe G. D. , and Humphries S. E. , Interleukin-6 Gene -174g>c and -572g>c Promoter Polymorphisms Are Strong Predictors of Plasma Interleukin-6 Levels After Coronary Artery Bypass Surgery, Arteriosclerosis, Thrombosis, and Vascular Biology. (2001) 21, no. 9, 1458–1463, 11557672. 11557672 10.1161/hq0901.094280

[27] Paparella D. , Semeraro F. , Scrascia G. , Galeone A. , Ammollo C. T. , Kounakis G. , de Luca Tupputi Schinosa L. , Semeraro N. , and Colucci M. , Coagulation-Fibrinolysis Changes During Off-Pump Bypass: Effect of Two Heparin Doses, Annals of Thoracic Surgery. (2010) 89, no. 2, 421–427, 10.1016/j.athoracsur.2009.10.041, 2-s2.0-74549192906, 20103314. 20103314

[28] Herron G. C. and Bates E. R. , Review of the Ticagrelor Trials Evidence Base, Journal of the American Heart Association. (2024) 13, no. 11, e031606, 10.1161/JAHA.123.031606. 38804216 PMC11255623

[29] Akkaif M. A. , Ng M. L. , Sk Abdul Kader M. A. , Daud N. A. A. , Sha′aban A. , and Ibrahim B. , A Review of the Effects of Ticagrelor on Adenosine Concentration and Its Clinical Significance, Pharmacological Reports. (2021) 73, no. 6, 1551–1564, 10.1007/s43440-021-00309-0, 34283374. 34283374

[30] So D. Y. F. , Wells G. A. , Lordkipanidzé M. , Chong A. Y. , Ruel M. , Perrault L. P. , le May M. R. , Sun L. , Tran D. , Labinaz M. , Glover C. , Russo J. , Welman M. , Chan V. , Chen L. , Bernick J. , Rubens F. , Tanguay J. F. , and RAPID CABG Investigators , Early vs Delayed Bypass Surgery in Patients With Acute Coronary Syndrome Receiving Ticagrelor: The RAPID CABG Randomized Open-Label Noninferiority Trial, JAMA Surgery. (2025) 160, no. 4, 387–394, 10.1001/jamasurg.2024.7066, 39969871. 39969871 PMC11840690

[31] Chanfreau-Coffinier C. , Friede K. A. , Plomondon M. E. , Lee K. M. , Lu Z. , Dinatale T. , DuVall S. , Vassy J. L. , Waldo S. W. , Cleator J. H. , Maddox T. M. , Rader D. J. , Assimes T. L. , Damrauer S. M. , Tsao P. S. , Chang K. M. , Voora D. , Lynch J. A. , Giri J. , VA Million Veteran Program , and Tuteja S. , CYP2C19 Polymorphisms and Clinical Outcomes Following Percutaneous Coronary Intervention in the Million Veteran Program, Clinical Pharmacology and Therapeutics. (2025) 118, no. 4, 876–884, 10.1002/cpt.3741, 40459439. 40459439 PMC12439012

[32] Jiang Q. , Yu T. , Huang K. , Huang X. , Zhang Q. , and Hu S. , The Impact of Medical Insurance Reimbursement on Postoperative Inflammation Reaction in Distinct Cardiac Surgery From a Single Center, BMC Health Services Research. (2022) 22, no. 1, 10.1186/s12913-022-07920-8. PMC9008956 35418067

[33] Higgs M. , McDonagh J. , and Sim J. , Clinical Practices for Defining, Detecting, and Diagnosing Postoperative Atrial Fibrillation After Coronary Revascularization Surgery - A Scoping Review, Australian Critical Care. (2025) 38, no. 1, 101083, 10.1016/j.aucc.2024.06.006. 39060153

[34] Sohn S. H. , Kang Y. , Kim J. S. , Paeng J. C. , and Hwang H. Y. , Impact of Functional vs Anatomic Complete Revascularization in Coronary Artery Bypass Grafting, Annals of Thoracic Surgery. (2023) 115, no. 4, 905–912, 10.1016/j.athoracsur.2022.10.029, 36334649. 36334649

[35] Hall R. , Majumdar M. , Cassidy R. , Feldman Z. , Suarez S. , Goudot G. , Bellomo T. , Jessula S. , Kirshkaln A. , and Dua A. , Use of Thromboelastography With Platelet Mapping to Identify Prothrombotic Coagulation Profiles in Patients With History of Cardiac Intervention Undergoing Lower Extremity Revascularization, Journal of the American College of Surgeons. (2023) 236, no. 3, 495–504, 10.1097/XCS.0000000000000497, 36729802. 36729802

